# Inhibitory control in teleost fish: a methodological and conceptual review

**DOI:** 10.1007/s10071-024-01867-5

**Published:** 2024-03-26

**Authors:** Tyrone Lucon-Xiccato

**Affiliations:** Department of Life Sciences and Biotechnology, University of Ferrara, Ferrara, Italy

**Keywords:** Cognitive control, Executive functions, Fish cognition, Individual differences, Inhibition

## Abstract

Inhibitory control (IC) plays a central role in behaviour control allowing an individual to resist external lures and internal predispositions. While IC has been consistently investigated in humans, other mammals, and birds, research has only recently begun to explore IC in other vertebrates. This review examines current literature on teleost fish, focusing on both methodological and conceptual aspects. I describe the main paradigms adopted to study IC in fish, identifying well-established tasks that fit various research applications and highlighting their advantages and limitations. In the conceptual analysis, I identify two well-developed lines of research with fish examining IC. The first line focuses on a comparative approach aimed to describe IC at the level of species and to understand the evolution of interspecific differences in relation to ecological specialisation, brain size, and factors affecting cognitive performance. Findings suggest several similarities between fish and previously studied vertebrates. The second line of research focuses on intraspecific variability of IC. Available results indicate substantial variation in fish IC related to sex, personality, genetic, age, and phenotypic plasticity, aligning with what is observed with other vertebrates. Overall, this review suggests that although data on teleosts are still scarce compared to mammals, the contribution of this group to IC research is already substantial and can further increase in various disciplines including comparative psychology, cognitive ecology, and neurosciences, and even in applied fields such as psychiatry research.

## Introduction

In many situations, humans withhold actions and thoughts that are motivated from inside or lured from an external stimulus, in order to reach a specific goal or to focus on a certain task. Examples include giving up a delicious but unhealthy dish for a diet, or focusing on homework while avoiding the distraction from more attractive activities. Another well-studied example is when one gives up a small, immediate reward (e.g., drinking a glass of wine) to obtain a large reward delayed in time (e.g., driving home safely; Logue 1988). Psychology researchers generally refer to the construct involved in these forms of behavioural regulation as inhibitory control (IC; reviewed in Diamond 2013). IC is generally associated to the core functions of our cognitive control, i.e., the executive functions, which enable the precise control and modulation of cognitive processes and, consequently, behaviour (Diamond 2013). It is worth noting that the exact structure of IC is still debated. Some authors tend to use IC as an umbrella term, recognising that different forms of inhibition are attributable to different constructs. For instance, the term ‘response inhibition’ (also referred to as ‘motor inhibition’ or ‘behavioural inhibition’) is often used when the a prepotent motor response is blocked; conversely, ignoring distracter stimuli is usually considered ‘attentional inhibition’ (Tiego et al. 2018). Another form of IC recognised by many authors is the one involved in the choice between present-small rewards versus delayed-large rewards. This is usually referred to as ‘self-control’ (e.g., Beran 2015; Kabadayi et al. 2016). For the scope of this review, I will primarily use the term IC to include all these facets, providing more details if necessary.

Behaviours that can be explained by IC have been also observed in non-human animals. Initial reports concern observations of animals’ natural behaviour. Ambushing predators are likely to exert IC when they detect a prey but wait until it gets close enough before attacking (e.g., Harper and Blake 1991; Pritchard 1965). Prey may use IC when inhibiting highly motivated activities such foraging or mating after detecting a predator (Ryer and Olla 1991). In hierarchical societies, subordinate individuals inhibit their tendency to consume a newly discovered food source in the presence of dominant individuals to avoid punishment (Whiten and Byrne 1988). These observations may reflect the effects of various independent cognitive mechanisms such as association learning and decision-making; however, they might be also explained by non-human animals exerting inhibition. After these early observations, clearer evidence was not available until the 1990s, when the IC of non-human species began to be routinely investigated with controlled laboratory tests. The literature now includes dozens of studies in various species of mammals and birds (e.g., Amici et al., 2009; Kabadayi et al. 2016; MacLean et al. 2014), and a few in reptiles (e.g., Szabo et al. 2020).

**Fig. 1:**
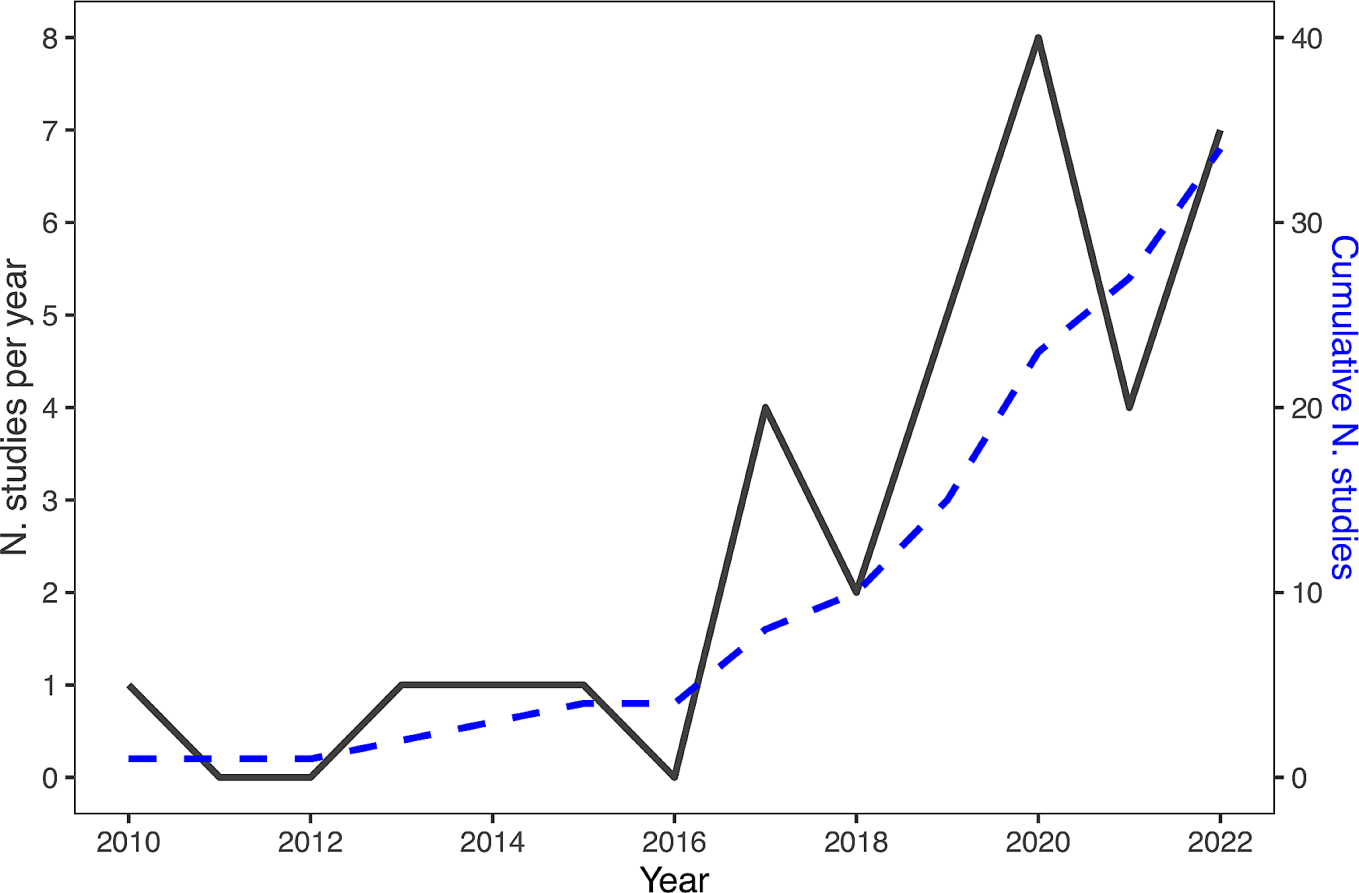
Trend of publications on inhibitory control of fish. The black line represents the number of studies published in each year and the blue dotted line represents the cumulative number of studies in the literature. Studies published in 2023 are not included in the plot

Regarding teleost fish, the literature on IC is relatively recent, with the first study published in 2010. However, studies of IC in fish have grown rapidly in the last five years (Fig. 1). In this work, I review the current knowledge on IC in teleost fish. The relevant articles were retrieved on Scopus and Google Scholar using keywords searches (e.g., ‘inhibitory control’ + ‘fish’, ‘inhibition’ + ‘fish’, ‘cylinder task’ + ‘fish’) and checking references and citations of the articles retrieved. First, I review the methodologies available to study IC in this group. Subsequently, I analyse the two main lines of investigation: comparative research aimed at investigating interspecific variation and intraspecific research dealing with variation due to factors such as sex, personality, genetic, and age.

## Inhibitory control paradigms for fish

Efficient paradigms are a critical prerequisite for cognitive research in animals, but they are often difficult to develop due to several problems. First, the species under investigation might present physical and behavioural constraints preventing the administration of certain tasks. This problem is particularly marked in fish, which have a peculiar morphology and habitat compared to most vertebrates. In this regard, however, research on animals’ IC has enjoyed a significant advantage. Humans’ IC deficits often appear early during ontogeny, leading psychologists to develop simple paradigms for children and even infants. Most of these simple paradigms could be adapted to other animal species, including fish. A second challenge is that the cognitive performance is measured indirectly, inferred from the subjects’ behaviour in the experimental setting. Accordingly, it is possible that performance is actually affected by non-target cognitive functions (Völter et al. 2018) and by non-cognitive factors (Rowe and Healy 2014). For instance, if a subject is rewarded with food to complete a task, hunger might be an important determinant of its performance. The attempts to exclude these factors are also dangerous because they might decrease the natural relevance of the task. One last critical challenge is that small variations in methods can severely affect the assessment of cognition and prevent comparisons among studies conducted in different laboratories without methodological standardisation (e.g., Prétôt et al., 2016).

In this section, I describe the main behavioural paradigms available for studying IC in fish and analyse them in the light of the aforementioned issues (Table 1). The paradigms will be operationally separated in two categories: those based on the spontaneous behaviour showed by fish in the experimental context and those based on training procedures that elicit specific responses via conditioning processes. Each of these two categories has specific advantages and limitations, which are also discussed in relation to the study’s objectives. For instance, spontaneous behaviour paradigms usually have higher ecological significance compared to training paradigms but are more susceptible to the influence of non-cognitive factors (Agrillo and Bisazza 2014). Another goal of this section is to suggest common guidelines to try to standardise the tests across different laboratories.

**Table 1:** Summary of the main paradigms used to assess IC in fish and the analyses on their advantages (+) and limitations (-)

Task	Type	Rapidity / simplicity	Ecological relevance	Confounding factors	Comparative data in fish
Barrier	Spontaneous behaviour	++	++	- -	+ +
Cylinder	Training	- -	-	-	+ +
Tube	Spontaneous behaviour / training	+	+	+	-
Reverse-reward contingency	Training	- -	- - (for cleaner wrasse: ++)	lack of data	- -
Delayed gratification	Training	- -	-	lack of data	+
Five-choice serial reaction time	Training	- -	-	+	-

### Barrier task

**Fig. 2:**
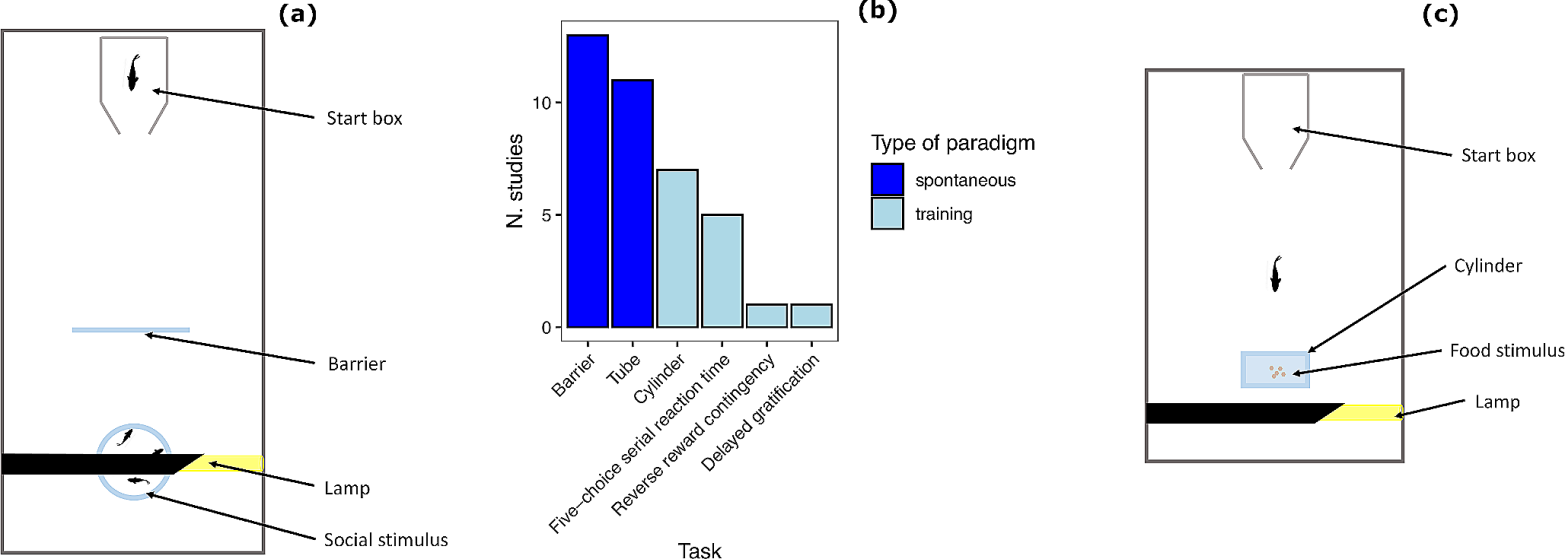
Main paradigms used to study inhibitory control in fish. **a** Diagram of the experimental apparatus used for the detour task. **b** Number of published studies for each inhibitory control task. **c** Diagram of the experimental apparatus used for the cylinder task

The barrier task is a relatively simple paradigm based on the spontaneous detour behaviour of the fish. It involves placing a barrier to be detoured between the subject and a desired goal (Fig. 2a). For this reason, it is also referred to as the detour task. I will use the term barrier task to avoid confusion because the following paradigm to be discussed (the cylinder task) also requires the detour behaviour. Several studies have exploited the barrier paradigm in comparative cognition research (reviewed in Kabadayi et al. 2018), although often with the scope of research being other than studying IC. For instance, the barrier task has been largely exploited to study lateralisation (Facchin et al. 1999) and mental representation abilities (Sovrano et al. 2018). In these cases, the barrier is made visible, for instance, with a grid or a fence, so that the subject can see it and recognise the need to perform the detour. To measure IC, the barrier is made of transparent material. Therefore, the subject is expected to be strongly lured by the sight of the goal and remain blocked by the barrier while trying to approach. By inhibiting the tendency to move directly towards the goal and moving away from it (e.g., turn to the left or to the right), the subject can successfully perform the detour. This task likely measures a form of response inhibition. In most of the studies in fish, the goal consists of a shoal of conspecifics placed in an adjacent tank or in a compartment of the testing tank (Lucon-Xiccato et al. 2017; Gatto et al. 2018; Santacà et al. 2019b; Wallace et al. 2020; Fig. 2a). As many small fish species exhibit a strong social attraction in novel situations, the barrier task usually takes place in a testing tank that is unfamiliar to the subject.

The barrier task allows the researcher to measure IC performance using at least two variables. The first variable is the time necessary to reach the goal, typically calculated from when the fish exits a start box to when it reaches the conspecifics. Alternatively, it can be measured as the time spent trying to pass through the barrier. If the subject undergoes multiple trials, the time variables usually decrease progressively (Lucon-Xiccato and Bisazza 2017b). Repeating the barrier trials makes it also possible to score a second variable based on whether the fish touched or avoided the barrier (Lucon-Xiccato et al. 2017). The subjects are indeed expected to contact the barrier during the initial trial and eventually stop doing so in latter trials.

Detouring an obstacle in the sense of moving away from the goal direction is a task that many species likely encounter in their natural environment, such as when navigating around vegetation, rocks, and other objects (e.g., Kimchi and Terkel 2003). While these natural obstacles are generally opaque, some, such as aquatic weeds, may permit partial visibility of the goal. Accordingly, an advantage of the barrier task is that it possesses some ecological relevance because it is based on spontaneous behaviour. Another strength of the barrier task is that it does not require training and is very quick. For these reasons, the barrier task is the most commonly used in fish research (Fig. 2b), and data useful for comparative research are available for many species (Kabadayi et al. 2018). However, the advantages of the barrier task are counterbalanced by the typical drawbacks of paradigms based on spontaneous behaviour. Performance in the barrier task may be affected by non-cognitive factors, such as subjects’ social motivation. Highly social individuals or species are expected to find detouring the barrier more difficult, all else being equal. Other cognitive functions may be also involved. For instance, the subject needs to maintain the mental representation of the goal and its spatial position to correctly perform the detour (Regolin et al. 1995; Sovrano et al. 2018). Moreover, when the detour is conducted over multiple trials, the resulting habituation to the testing environment is expected to decrease social attraction, indirectly affecting the inhibitory performance. This poses a limit to the number of trials that can be administered to an individual subject. The problem of sociability may also concern the use of the barrier task in applied research, such as the study of psychoactive drugs, which often alter individuals’ sociability (e.g., Brodin et al. 2013). An interesting solution to these issues could be the use of a food reward adopted by Brandão and colleagues (2019) in cichlid fish. Attraction towards food also varies with some extent according to intrinsic and extrinsic factors, but at least is not expected to change substantially over multiple, well distanced trials. Another limitation or confounding factor is that some species may respond to olfactory cues when the chamber with the social stimuli is connected to the compartment with the focal individual (Santacà et al. 2019b). This problem can be reduced with stimulus chambers that are completely isolated, with the cost that the social reward will then have no odour, and chemical cues are known to contribute to interaction between conspecific fish (Solomon 1977).

### Cylinder task

The cylinder task is a paradigm based on a training procedure commonly employed to study IC in mammals and birds (Kabadayi et al., 2020; MacLean et al. 2014). The goal is a small amount of food placed inside a transparent cylinder, with the cylinder laying horizontally on the bottom of the apparatus (Lucon-Xiccato et al. 2017; Fig. 2c). The two open sides of the cylinder are perpendicular to the position of the subject at the beginning of trial. Therefore, to solve the task, the animal has to perform a detour, entering the cylinder laterally, without touching its surface. A second version of the cylinder task has the cylinder placed vertically, with only one available opening on the top (Minter et al. 2017; Keagy et al. 2019). The primary distinction between the barrier task and the cylinder task is that complexity of the detour behaviour necessary to solve the cylinder task requires a training phase before IC performance may be measured. In this training phase, the subject is usually habituated to find the food inside an opaque cylinder and develops the motor sequence necessary to enter the cylinder. Following the initial training phase, the opaque cylinder is replaced with the transparent cylinder and the fish’s performance is recorded for numerous trials (e.g., 50). The dependent variables usually collected are the percentage of correct responses in which the fish enters the cylinder without touching it and the time required to enter the cylinder.

The main limitations of the cylinder task are the time required to train the subjects and the complexity of the apparatus. Completing an experiment (e.g., training phase plus test phase) may require up to 20 days for a single subject. The length of the experiment makes it more practical to perform in the subject’s home tank; however, several apparatuses, such as a start box and guillotine door, need to be added to permit the experimenter to set the cylinder without interference from the subject. These complex apparatuses make the setting very different from natural situations. While probably not as susceptible as the barrier task, the cylinder task is also influenced by non-cognitive factors and other cognitive functions. Recent studies with birds have raised concerns about the effects of motivation, body condition (van Horik et al. 2018) and learning (Kabadayi et al. 2017b). In guppies, *Poecilia reticulata*, the performance in the cylinder task has been shown to be quite robust to differences in subjects’ experience. Santacà and colleagues (2019a) raised guppies in glass aquaria, control aquaria with opaque walls, or aquaria with transparent obstacles to test the effect of experience on the cylinder task performance. The guppies’ performance was not affected by the treatment condition. Moreover, Santacà et al. (2019a) conducted an experiment with a cylinder pierced with small holes to allow the food cue to permeate. They found no effects of this modified cylinder on guppies’ performance, suggesting that the task is robust to olfactory cues (Santacà et al. 2019a). The effects of other potential confounding factors remain to be investigated. Nevertheless, the cylinder task bears an important comparative advantage because the literature contains data for a large number of mammalian and avian species (e.g., MacLean et al. 2014).

### Tube task

The tube task is a paradigm based on spontaneous feeding behaviour of fish that was developed in my laboratory following a paradigm used in cuttlefish research (Agin et al. 1998). The subject is exposed to a laboratory glass tube containing live prey such as *Artemia salina* nauplii, which are commonly provided to fish as food in most facilities (Lucon-Xiccato and Bertolucci 2019). Fish are expected to attempt to directly reach the prey, but instead they come into contact with the glass, a behaviour that can be quantified precisely with recordings played back on a computer. After each attempt to reach the prey, the subject can either perform another attempt or inhibit the foraging behaviour, the latter determining a reduction in the number of attempts over time. This reduction may arise by multiple mechanisms, including IC, habituation learning, and association learning. The second mechanism does not fully apply because there is not active stimulation of the sensory system (Rankin et al. 2009). Moreover, it was found that increasing number of prey, which corresponds to increasing the stimulations, did not accelerate the decrease in the number of attempts, as expected for habituation mechanisms (Rankin et al. 2009). The contribution of associative learning mechanisms is more difficult to rule about, but this is also true for other inhibitory tasks (e.g., the fish can learn to detour around the barrier). However, in a recent study, it was found that the tube task yields results comparable to that of the cylinder task (Montalbano et al. 2020). This suggests a common underlying cognitive function (e.g., IC) explaining performance in both tasks.

The tube task was developed to exploit spontaneous behaviour similar to the barrier task, making it far more efficient than the cylinder task for screenings of large populations. However, although in theory the response to the prey is spontaneous, we found that the likelihood of response increases if the subjects underwent a short training phase (2–3 days) for feeding in the area of the apparatus where the tube is presented in the test phase. Consequently, the time and the effort required to perform the tube task are slightly greater than those for the barrier task, although still substantially smaller compared to pure training paradigms. Additionally, the tube task was developed to avoid the confounding effects of sociability described for the barrier task and interferences of olfactory cues since the stimulus is sealed in the tube (Santacà et al. 2019a). A methodological issue is that the performance is measured as a change in behaviour over time, often complicating the statistical approach when comparing groups. However, by selecting an appropriate testing time, differences in IC can be scored using the overall number of attempts.

### Reverse reward contingency task

The reverse reward contingency task is based on training and is commonly adopted in comparative research in mammals and birds (e.g., Anderson et al. 2000; Jelbert et al. 2016). A difference from the tasks mentioned above is that it does not rely on transparent objects, but on stimuli with different values. The subject is presented with a dichotomous free choice between a larger and a smaller reward, usually triggering the choice for the larger reward. However, the experimenter manipulates the accessibility to the rewards: if the subject chooses the larger reward, it obtains the smaller reward, and vice versa. Therefore, to obtain the larger reward, the subject has to inhibit its attraction towards the larger reward. This task might depend on both response and attentional inhibition.

This method was applied only once to fish. Danisman and colleagues (2010) presented to their subjects the choice between 4 versus 1 pieces of smashed shrimp and, in a modified version of the task, the choice between the large food reward and no food. The reverse reward contingency task presents the value of being exploited in various other vertebrate groups. One limitation is related to the fact that the paradigm requires an extended training procedure before observing the expected response, i.e., initially, the subject is be attracted by the larger, incorrect reward. Moreover, excluding a few species with peculiar biology, the choice for the smaller reward is unnatural.

### Delayed gratification task

The delayed gratification describes various versions of a paradigm that putatively measures self-control and similar skills (e.g., patience) (e.g., Duckworth et al. 2013; Evans et al. 2012; Parrish et al. 2014). Self-control is considered the capacity to resist a small, immediate reward in order to obtain a larger reward later in time (Beran 2015). Critically, it is a form of inhibition that requires an active decision between the two rewards. The general procedure of the delayed gratification task involves presenting the subject with an immediate, low-value reward must be ignored to obtain a reward with larger value at a later moment. The delayed gratification task is therefore similar to the reverse reward contingency task but implies the temporal factor typical of self-control.

This paradigm has been applied to fish with variations in the type of stimulus (e.g., larger versus smaller food; favourite versus less favourite food; Aellen et al. 2021). While the delayed gratification task requires a relatively complex training procedure and is somehow unnatural (Aellen et al. 2021), it offers the possibility of being compared with a large literature in mammalian and bird species (e.g., Duckworth et al. 2013; Evans et al. 2012; Parrish et al. 2014). It may become important in fish research because it is the only paradigm used in this group to measure the temporal aspects of IC (Beran 2015).

### Five-choice serial reaction time task

The five-choice serial reaction time task is a training paradigm used in pharmacology research with rodent models (Chudasama and Robbins 2004; Grottick and Higgins 2000; Sanchez-Roige et al. 2012). Parker and co-authors (2013) developed a version of this paradigm for fish, based on a five-chamber apparatus. The subject has to learn to enter a chamber briefly indicated by a cue (a small light) to obtain a food reward. The dependent variable related to inhibition is the anticipatory errors of the fish, which are scored when the fish visit a chamber in the inter-trial interval before the system indicates the correct chamber. Anticipatory errors are considered at least in part due to failures in inhibiting behaviour, probably involving both response and attentional inhibition. However, some authors attribute them to attention and impulsivity (Fletcher et al. 2013; Robbins 2002).

An advantage of this task is that it is commonly used in laboratory rodents (e.g., Chudasama and Robbins 2004; Grottick and Higgins 2000; Sanchez-Roige et al. 2012). Therefore, results in fish can be analysed and interpreted based on an extensive literature. A potential limitation of the task is that it requires training and a complex apparatus, resulting in limited interpretability in terms of behaviour expressed by the species in nature. Among the advantages, it should be noted that Brock and colleagues (2017) developed an automated training chamber to administer the task, enabling screenings of relatively large subject populations (Faillace et al. 2018; Parker et al. 2014, 2015).

## Comparative research on fish inhibitory control

Much of the research on animals’ IC is aimed at comparing different species and ultimately, understanding the evolutionary causes of interspecific differences (e.g., Amici et al. 2008; MacLean et al. 2014). The same aim has driven several studies in fish. One line of research in fish focussed on the hypothesis of ecological specialisation of IC, whereas a second line of research was stimulated by the debate on the role of a species’ brain size on cognition. Moreover, several studies have assessed the effects of various task parameters on the performance to understand the factors explaining interspecific differences and eventual similarities in the mechanisms underlying IC.

### Ecological specialisation of inhibitory control

A set of studies have focussed on the cleaner wrasse, *Labroides dimidiatus*. The interest on this species is mainly driven by its peculiar foraging behaviour, which involves a fascinating example of interspecific mutualism. Cleaner wrasses provide a service to heterospecific ‘client’ fish by eating their ectoparasites. Although this biological interaction is mainly based on cooperation, cleaner wrasses prefer to eat the mucus of the client fish. When they do so, the client flees because the mucus has important protective functions (e.g., Dash et al. 2018). To avoid losing clients, the cleaner wrasses must refrain their preference to consume the mucus and exercise inhibition/self-control. It is expected that the cleaner wrasse might have evolved enhanced inhibitory abilities in the context of foraging. Danisman and co-authors (2010) tested this hypothesis with a reverse reward contingency task, but none of the eight subjects learned to solve the task, even after an extended training consisting of 200 trials. Although this early study did not support ecological specialisation of IC in the cleaner wrasse, it should be said that the task adopted is challenging even for primates (Boysen and Berntson 1995).

A more recent study has provided further insights on cleaner wrasses’ IC. Aellen and colleagues (2021) exposed the fish to a delayed gratification task with stimuli similar to those used in the reverse reward contingency task (i.e., small versus large number of food items). The subjects successfully solved the task, with performance not substantially different from that observed in primates (Aellen et al. 2021). Considering that the main difference between the delayed gratification and the reverse reward contingency task is the timing of the reward, it is possible that cleaner wrasses perform poorly when the highly valuable reward is visible at the moment of the choice (but see Prétôt et al. 2016b). Interestingly, other wrasse species were also observed in the delayed gratification task, including the cleaner species *L. bicolor* and the non-cleaner species *Halichoeres trimaculatus* and *H. hortulanus*. At least one of the non-cleaner wrasses performed well in the task, which is again against the general hypothesis of an ecological specialization of IC in this group.

In conclusion, there is no strong support for the hypothesis of ecological adaptation of cleaner wrasses. However, the data seems far from conclusive, as they are based on few subjects and few species. Additionally, part of the studies has been performed with the delayed gratification task, which may not fully represent the situation of a cleaner wrasse-client interaction where both the preferred (i.e., mucus) and the non-preferred food (i.e., ectoparasites) are simultaneously available. It is worth mentioning that other studies have reported high performance of cleaner wrasses in the ephemeral reward task, often superior to the performance observed in primates, rats, and pigeons (Prat et al. 2022; Prétôt et al. 2016a, b). This task requires the subject to choose between two rewards, one of which can be always accessed, while the other disappears after choosing the alternative reward. Although this task was not aimed to measure IC, it has been proposed that the ability to inhibit impulsive choices is involved (Zentall and Case 2018). The application of more paradigms to the system is certainly necessary.

A second line of investigation on ecological specialisation of IC is related to the social system. Theory suggests that inhibition is an important ability to live in social systems, especially when the interactions between individuals are complex. For instance, the adaptive social response varies depending on the social context, such as the presence of dominant or subordinate group mates. This requires the individuals to change continuously their behaviour according the group mates, often withholding responses that are appropriate in other contexts (Aureli et al. 2008). A comparative study in primates supported this hypothesis, revealing greater IC in species with more complex social systems (Amici et al. 2008). One study tested this hypothesis in six species cichlid fish with different social system (Salena et al. 2022). The species observed belong to the Lamprologini tribe, which has independently evolved group-living and cooperative breeding five times. Three of the species observed (*Neolamprologus pulcher*, *N. multifasciatus*, and *Julidochromis ornatus*) display cooperative breeding, whereas the remaining three species (*Telmatochromis temporalis*, *Altolamprologus compressiceps*, and *N. tretocephalus*) are considered non-cooperative breeders. The test adopted involved a transparent partitioning similar to that described for the barrier task. By observing the tendency to attempt swimming through this transparent barrier, the authors compared the IC of the six Lamprologini cichlids, finding no difference ascribable to the social system. Interestingly, another study (discussed in Sect. 4.5) reported that social environment affected IC within-species via plasticity (Lucon-Xiccato et al. 2022a). The same may occur in cichlids, calling for further investigations on this potential ecological specialisation of IC.

### Brain size and inhibitory control

**Fig. 3:**
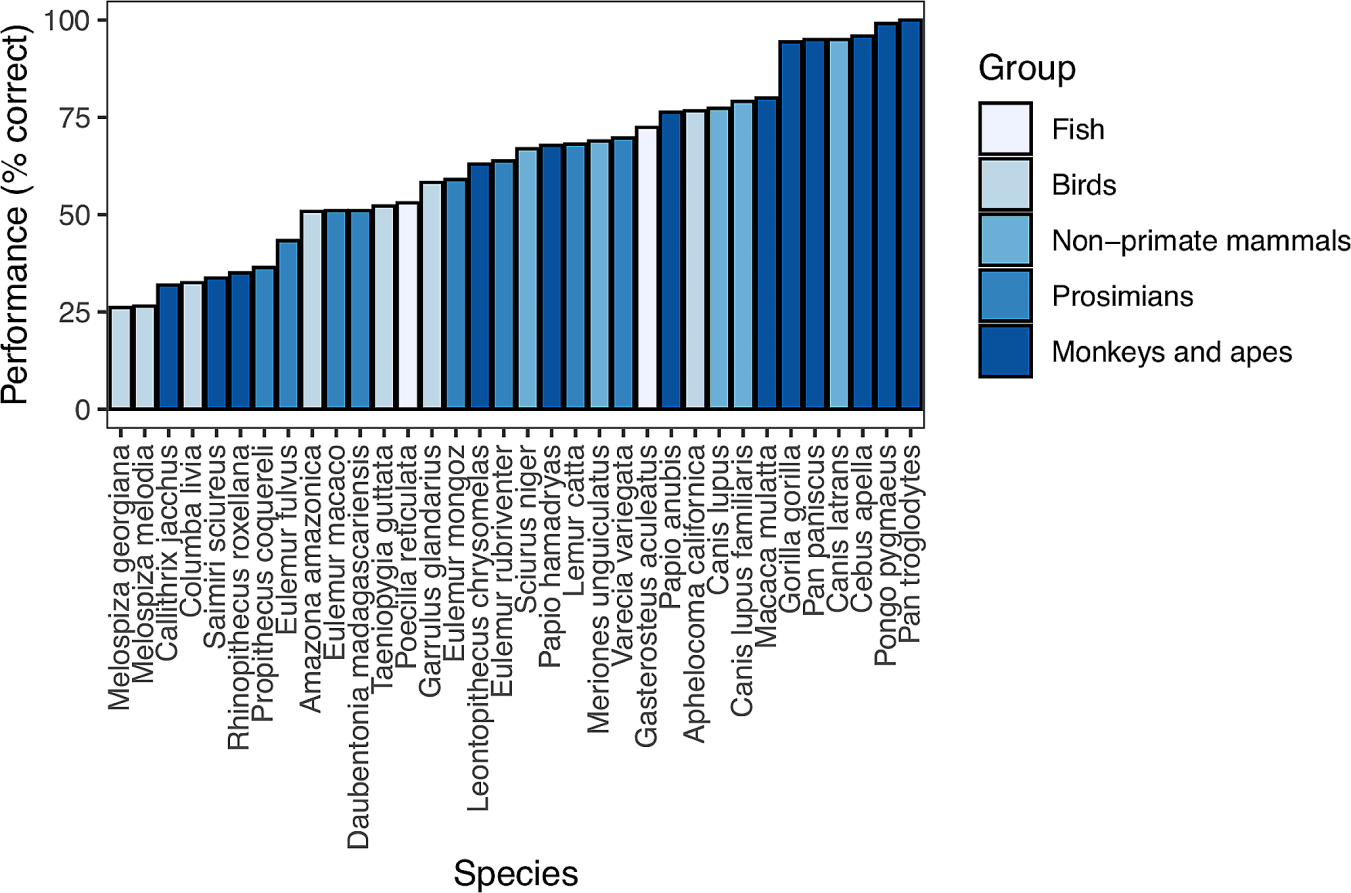
Comparison of performance in the cylinder task between teleost fish, birds, and mammals. Data of fish retrieved from Lucon-Xiccato et al. (2017) and Minter et al. (2017); data of birds and mammals retrieved from MacLean et al. (2014). Bars represent mean percentage of correct trials obtained from the first 10 trials of the experiment in all of the species, except for the sticklebacks (first four trials). Colours indicate main taxonomic group

A line of research on fish IC has focussed on its evolutionary link with brain size. It has been traditionally believed that species with larger brain size display greater cognitive abilities (reviewed in Jerison 2012), although current knowledge does not support the generality of this relationship (Healy & Rowe, 2013; Herculano-Houzel 2017; Perry et al., 2017). For instance, the literature on fish includes a long list of unexpected skills in small species, in spite of their small brain size among vertebrates (reviewed in Bshary and Brown 2014; Pouca and Brown 2017). A large comparative study with the cylinder task (and a second task not yet adopted in fish, the A-not-B task) investigated the relationship between brain size and IC in mammals and birds, finding a positive correlation between a species’ inhibitory performance and its absolute brain size (MacLean et al. 2014). If this relationship held true across the entire vertebrate clade, fish would be expected to exhibit low IC due to their small brain size. An adaptation of the cylinder task (Lucon-Xiccato et al. 2017) compared the performance of guppies with that of the mammals and birds observed in the study by MacLean and co-authors (2014). Guppies successfully learned the task, achieving a performance indistinguishable from that of most mammals and birds (Fig. 3). The only species that clearly outperformed the guppies were monkeys and apes in the study by MacLean and co-authors (2014) and corvids in a later study (Kabadayi et al. 2016). An even higher performance was obtained by sticklebacks, *Gasterosteus aculeatus*, observed in a modified version of the cylinder task (Fig. 3). These data are against the generality of the large brain-high IC association.

A concern raised in the guppy study was that, like most laboratory fish, the subjects spent their lives in glass aquaria. It was suggested that this experience with transparent surfaces might have favoured their ability to handle the transparent cylinder. A test of this idea found no substantial support: guppies reared in standard aquaria and guppies raised in aquaria with opaque walls did not differ in their performance in the cylinder task (Santacà et al. 2019a). These studies allow for the conclusion that the gap between fish and warm-blooded vertebrates in the cylinder task is not proportional to their difference in brain size. Indeed, the average size of a guppy’s brain is at least 100 times smaller than that of the species tested by MacLean and colleagues (2014).

An explanation for the whole pattern of results on IC and brain size in vertebrates would be that interspecific differences in IC are related to different brain areas. This idea is supported by a recent artificial selection study in guppies. Triki and colleagues (2022) selected via controlled breeding a line of guppies with large telencephalon compared to whole brain size and a line of guppies with small relative telencephalon. Despite significant anatomical differences, such as the absence of a cortex, the telencephalon in fish is generally considered homologous to that of mammals and birds (Briscoe and Ragsdale 2019). This link is particularly evident looking at its functions. In fish, the telencephalon involves multimodal sensory integration, cognitive processing, and behavioural control (Calvo and Schluessel 2021; Flood et al., 1976). The line of guppies with large telencephalon outperformed the line with small telencephalon in a detour task, indicating that in fish, the size of specific brain areas might be more important than the overall brain size in determining IC. Other explanations for the interspecific differences in the link between brain size and IC remain untested. First to be mentioned, the issues on the differential response of distantly-related species to the same task (Sect. 3.3) and a potential ecological specialisation of IC in guppies.

### Factors affecting inhibitory control

Comparing the performance of different species in cognitive tasks is generally acknowledged as problematic due to the influence of various non-cognitive factors (e.g., Prétôt et al. 2016a, b). This issue has emerged for IC tasks based on the response to a transparent obstacle, such as the barrier or the cylinder task. For example, in a comparison of 4 teleost fish with the barrier task, it was found that one species, the zebrafish *Danio rerio*, substantially outperformed the others (*Poecilia reticulata*, *Xenotoca eiseni*, *Oryzias sarasinorum*; Santacà et al. 2019b). However, control tests revealed that the higher performance of the zebrafish was due to the sensory modality used to deal with the task, rather than to heightened IC per se. Indeed, zebrafish, unlike the other three species, performed the detour following the olfactory cues from the stimulus shoal. This cue could not pass through the barrier and if followed led the fish to execute a perfect detour. Therefore, species differences in preference for visual versus olfactory cue can lead to erroneous conclusions on IC, an issue that likely affected also studies in mammals and birds (e.g., MacLean et al. 2014).

An alternative strategy to compare species’ IC might be to examine the mechanisms rather than the performance itself. This can be easily done by altering parameters potentially important for the task and observing the change in performance. Several studies on guppies and cleaner wrasses provide data that can be interpreted with this approach. The stimulus used as goal is one of the most interesting parameters in this type of studies: in mammals, including humans, inhibition is more difficult when the goal is closer (Diamond and Gilbert 1989; Junghans et al. 2016). Moreover, another factor known to affect inhibitory performance is the value of the reward: when the reward has a larger value individuals are more motivated to obtain it (Odum et al. 2006; Rosati et al. 2007). Guppies’ IC has been shown to be influenced by similar factors. The time necessary to solve the barrier task increased when the stimulus shoal was placed closer to the barrier (Gatto et al. 2018). Moreover, increasing the value of the reward in the tube task (i.e., adding more prey) resulted in difficulties in withholding the attack behaviour (Lucon-Xiccato and Bertolucci 2019), although the reward’s value did not alter the outcome of the barrier task (Gatto et al. 2018). Similar modulations based on reward type and value have been observed in delayed gratification tasks in primates and corvids (Dufour et al., 2007; Hillemann et al. 2014; Ramseyer et al. 2006). Cleaner wrasses also exhibited a facilitation effect when the difference between small and large quantity rewards increased (from 1 versus 2 items to 1 versus 8 items; Aellen et al. 2021). Interestingly, Aellen and colleagues (2021) also manipulated reward quality, requiring cleaner wrasses to inhibit choosing a non-preferred food type to obtain a preferred type, but the subjects failed this version of the task.

Last, an effect commonly reported in birds and mammals is the impact of experience with the task on IC performance. For instance, two primate species (*Saguinus oedipus oedipus*: Santos et al. 1999; *Pongo pygmaeus*: Vlamings et al. 2010) and four parrot species (*Primolius couloni*, *Ara glaucogularis*, *A. ambiguus* and *Psittacus erithacus*: Kabadayi et al. 2017b) have shown improvements over testing trials in their inhibitory performance. In working dogs, IC performance improves also in response to the training program (Barrera et al. 2019). This performance improvement is probably attributable to several, non-mutually exclusive mechanisms. First, IC might plastically increase with training (Hartmann et al. 2016). Second, IC performance might be more effective due to co-optation of other processes such as learning (Murray et al. 2005). Last, the subjects develop familiarity with the protocol that might indirectly improve performance. Two separate studies reported the same improvement effect with the detour task in guppies (Lucon-Xiccato et al. 2017; Gatto et al. 2018) and two studies detected it in various cichlid species (*Oreochromis niloticus*: Brandão et al. 2019; *Neolamprologus pulcher*, *N. multifasciatus*, *Julidochromis ornatus*, *Telmatochromis temporalis*, *Altolamprologus compressiceps*, and *N. tretocephalus*: Salena et al. 2022). At least at level of performance, fish’s IC appears to respond to training and experience similarly to what observed in other vertebrates, although the exact mechanism is not necessarily the same.

In conclusion, this record of studies suggests that despite challenges in directly comparing IC among species, valuable insights can be gained by examining the underlying mechanisms of performance. In several instances, findings obtained through this approach lend support to the notion of shared mechanisms across vertebrates, potentially stemming from either a conserved cognitive trait or convergent evolutionary processes.

## Within-species variability in inhibitory control of fish

A feature of IC that has long interested human psychologists is its variability. Psychometric experiments indicate that individuals differ consistently in their ability to inhibit, and that these differences are also related to genes (Crosbie et al. 2013). Remarkably, individual differences in inhibition bear consequences, such as reduction of cognitive capacities (Fuhs and McNeil 2013; Linck et al. 2012), scholastic success (Allan et al. 2014; Clair-Thompson and Gathercole 2006), and job achievements (Converse et al. 2014), suggesting an important role of IC variability in everyday activities. IC variability has been also reported consistently across a range of other mammalian species (Beran and Hopkins 2018; Gnanadesikan et al. 2020) and in birds (Davidson et al. 2022; Langley et al. 2020). Studies in fish have addressed the question of IC variation in various species and from various perspectives.

### Sex differences

A relatively large literature in fish has addressed sex differences in executive functions, including IC (reviewed in Lucon-Xiccato 2022). For example, comparisons between the two sexes with the barrier task found greater female performance in guppies (Lucon-Xiccato and Bisazza 2017b), in eastern mosquitofish (*Gambusia holbrooki*; Vinogradov et al. 2022), and in cleaner wrasses (Triki and Bshary 2021). A large sex difference in the same direction was found when guppies were presented with the tube task (Lucon-Xiccato et al. 2020b). In modified versions of the cylinder task, female Nile tilapias, *O. niloticus*, solved the barrier task significantly faster than males (Brandão et al. 2019), whereas male sticklebacks, *G. aculeatus*, showed greater inhibitory scores compared to females (Keagy et al. 2019). Interestingly, one study on the western mosquitofish, *Gambusia affinis*, (Wallace et al. 2020) and one study on the ocellated wrasse, *Symphodus ocellatus*, (Cummings et al. 2022) found no sex differences with the detour task.

The aforementioned studies offer initial evidence of variability within-species in IC. Comparing the two sexes is a simple experimental design to investigate cognitive variability, and this has certainly favoured this line of research. It is worth noting the substantial variability between species regarding the presence and the direction of the sex differences in IC. Part of this variability may be due to ecological and reproductive differences between the two sexes. Cognitive sex differences are indeed expected to evolve when the two sexes are required to solve different cognitive tasks or emit different behaviours (Jones et al. 2003; Lucon-Xiccato 2022). For instance, male sticklebacks exhibit extended parental cares for their offspring, refraining from eating the eggs and fry, possibly contributing to their greater IC performance compared to females (Keagy et al. 2019). In guppies, males display highly persistent courtship and harassing behaviour. This might be linked to their reduced IC performance relative to females (Lucon-Xiccato and Bisazza 2017b). Fish, with their sexual variability in behaviour (Magurran and Garcia 2000), are promising to understand the adaptive significance and the evolution of cognition in the two sexes. An additional intriguing aspect of fish is the presence of alternative mating tactics in several species. In one species, such tactics have already been associated to differences in IC (Cummings et al. 2022). Further investigations can provide valuable insights into the impact of sexual selection on cognition.

### Individual differences and fitness consequences

Individual differences represent a more subtle form of intraspecific variation in cognition, encompassing differences among individuals of the same sex. Identifying individual differences is a more complex task compared to discerning sex differences. A common approach to assess whether a cognitive (or behavioural) trait varies among individuals involves examining its repeatability (Rowe and Healy 2014). For individual differences with biological meaning, we expect (significant) repeatability in the rank order of individual performance across repetitions of the same task. For IC, this formal analysis has been conducted in guppies (Lucon-Xiccato et al. 2020b, c; Macario et al., 2020) and in zebrafish (Lucon-Xiccato et al. 2020c, d). These studies yielded significant evidence of repeatability in all but one case (guppies: Lucon-Xiccato et al. 2020c). The cause for this disparate result is unclear, as there are no apparent methodological differences compared to some of the other studies. It is essential to acknowledge that the repeatability method is susceptible to potential biases, as subjects might modify their behaviour across trials due to familiarization with the apparatus and the procedure. This could introduce variability that affects the repeatability estimation. Further research with diverse tests is crucial for a more comprehensive understanding of repeatability in IC.

An interesting finding of a study employing the tube task is that the individual differences were remarkable. Individuals exhibiting lower IC displayed performance levels approximately 300 times lower than their higher IC counterparts (Lucon-Xiccato et al. 2020b). This may be the largest cognitive difference between individuals observed in animals so far, making it important to analyse its evolutionary causes and fitness consequences. A further promising line of research for future studies concerns the molecular and neural mechanisms of such variation. The extent of variance observed in fish may be of help for this purpose. Additionally, it’s noteworthy that individual differences in guppies’ IC were consistent across different tasks, including the barrier task and the cylinder task (Montalbano et al. 2020). This consistency across tasks suggests the possibility of a single underlying function contributing to IC. It is essential to note that the presence of a single function for inhibitory control has not been universally supported by studies in other animals (e.g., Brucks et al. 2017), which warrants careful examination in fish.

Three studies involving sticklebacks have explored potential associations between individual differences in IC and reproductive fitness. In an initial investigation, it was observed that females exhibited a mating preference for males with higher IC (Minter et al. 2017). In contrast, male mating preference was not affected by females’ IC (Keagy et al. 2019). This suggested a reproductive fitness advantage of males, but not females, with greater IC. The observed female mate choice might have evolved because of indirect benefits: by choosing a male with higher IC, females might obtain reduced risk of cannibalism for their offspring. A third study reported a more complex pattern, which is currently difficult to interpret: only females with relatively low IC preferred males with high IC (Álvarez-Quintero et al. 2021). Moreover, evidence suggested that males with higher IC build more elaborated nests (Álvarez-Quintero et al. 2021), which can explain the mate choice observed in females. These records strongly suggest a link between an individual’s IC and fitness, at least in the context of reproduction. Future studies should address this effect in nonreproductive contexts as well. Given the considerable variability in IC observed in species such as the guppies (Lucon-Xiccato et al. 2020b), teleost fish might favour large leaps forwards in our understating of the fitness consequences of cognition.

### Covariance with personality and other cognitive traits

Various lines of evidence suggest that individual differences in cognitive traits are correlated with variability in behavioural traits, such as personality (Carere and Locurto 2011). Additionally, IC can be also associated with individual differences of other cognitive traits (e.g., Beran and Hopkins 2018). Initial investigations in guppies revealed that individuals with higher IC performance in the tube task were less active (Lucon-Xiccato et al. 2020c). Moreover, guppies with higher IC performance spent more time in the centre a novel environment. A similar relationship was found in zebrafish (Lucon-Xiccato et al. 2020c). Time spent in the centre of a novel environment is usually considered an indication of boldness, one of the main personality traits described in fish (Toms et al. 2010). A second study in guppies confirmed the positive association between IC and the time spent in the centre of a novel environment (Savaşçı et al. 2021). However, in this second study, behaviour in another test specifically developed to assess fish boldness (e.g., the scototaxis test: Maximino et al. 2010) did not relate to IC performance. This suggested that exploratory tendency, rather than boldness, might be associated to IC in guppies. High exploratory tendency might indeed cause individuals to spend more time in the centre of a novel environment as well. A study on sticklebacks also provided indirect evidence of covariance between IC and a measure of boldness recorded in the detour task (Keagy et al. 2019). However, another recent study with the same approach found only marginally significant evidence of this relationship in eastern mosquitofish (Vinogradov et al. 2022). While there seems to be general support for the covariation between personality and IC in fish, the specific personality traits involved are not clearly defined. It would be also interesting to understand whether the relationships vary across species and what are their evolutionary causes. For instance, if IC performance is positively associated with boldness and/or exploration, does this contribute to bolder individuals inhibiting antipredator behaviours and being more inclined to take risks in novel environments? Additional studies are needed to address this and similar questions.

Concerning the covariance between IC and other cognitive traits, one study reported a correlation with brain functional lateralisation (Lucon-Xiccato et al. 2020d). Lateralisation indicates the extent to which individuals process a type of information in each of the two brain hemispheres (Bisazza et al. 1998). In fish, it is easily measurable from eye preferences. Individuals that process a certain stimulus with the right hemisphere look at it preferentially with the left eye. In zebrafish, it was reported that with greater IC performance in the tube task processed social information mostly with the left hemisphere of the brain (Lucon-Xiccato et al. 2020d). In cleaner wrasse, IC measured with the detour task was negatively correlated with performance in a reversal learning task (Aellen et al. 2022). Reversal learning is usually considered a measure of another executive function, cognitive flexibility. However, there might be a direct contribution of IC. During the reversal learning experiment, the subject is first trained to select a stimulus between two options to obtain a reward. Then, the contingency is switched, requiring the fish to select the previously unrewarded stimulus. In this later phase, it is arguable that the subjects have also to inhibit the choice of the previously rewarded stimulus. Last, in guppies, Montalbano and colleagues (2020) found no significant association between IC and problem-solving (Montalbano et al. 2020), which is considered an executive function by some authors (e.g., Diamond 2013). Although preliminary, these findings suggest that studying covariation between IC and other cognitive functions can be attempted with greater extent in the future.

### Genetic differences

In humans, a significant proportion of individual variance in IC is heritable (Crosbie et al. 2013) and recent evidence suggests the same for nonhuman animals (Langley et al., 2021). In fish, direct investigations are still lacking, but several studies have provided indirect evidence of potential genetic effects on IC. One study on guppies compared individuals from two populations raised in similar conditions in the laboratory, detecting significant differences: individuals descending from a wild population of the lower Tacarigua River (Trinidad) outperformed individuals of a domesticated strain in the detour task (Gatto et al. 2018). Through an indirect comparison, it is also possible to appreciate differences between guppies of the aforementioned wild population and that of the lower Aripo River (Trinidad) tested by Macario and colleagues (2021). While the differences between domestic and wild populations might derive from relaxed selection in captivity, those between the two wild populations may be the result of local adaptation. The level of predation risk is a well-known driver of local adaptation in Trinidadian guppies (e.g., Gordon et al. 2017; Torres-Dowdall et al. 2012). However, both the lower Tacarigua River and the lower Aripo River are described as high predation risk sites, and guppies from the two sites have comparable antipredator responses (Magurran 2005). Guppies from the two drainages might also differ in foraging behaviour and risk of interspecific competition (Magurran 2005), suggesting that these factors should be investigated as drivers of inhibitory control differences. Further evidence of IC heritability derives from the study by Triki and colleagues (2022) in guppies: they found a significant response to artificial selection for different telencephalon size, which implies genetic differences that directly or indirectly determine variation in IC. Last, differences in IC with the tube task were also reported between two laboratory lines (AB and Tubingen) and one domestic strain obtained from a pet shop (Lucon-Xiccato and Bertolucci 2020).

Although the genetic bases of IC in fish have not been directly analysed so far, the studies reported above provide some indirect evidence. This evidence should be considered preliminary as based on comparisons between only a few different strains of fish. If future studies confirm the genetic bases of IC with more controlled experiments, it will be possible to use fish to investigate the selective pressures acting on IC (e.g., Huizinga et al. 2009). Moreover, the zebrafish may become interesting for identifying specific loci and polymorphisms associated with IC variability exploiting functional and quantitative genetic approaches (Wright 2011). Recently, promising results have been obtained by undertaking this task and generating a CRISPR/Cas9 zebrafish knockout for a gene controlling a neurotropic factor important for IC in rodent models. This mutant zebrafish displayed marked deficits in IC compared to the control group (Lucon-Xiccato et al. 2022b).

### Development and phenotypic plasticity

Human IC progressively increases during childhood (Williams et al. 1999), and similar developmental trajectories have been reported in other primates and in a bird species, the raven *Corvus corax* (Diamond 1990; Kabadayi et al. 2017). In fish, two different species have been assayed looking for developmental changes in IC. Savaşçı and colleagues (2021) studied guppies at the age of 10, 20, 40 and 90 days with the cylinder task, the latter age corresponding to the onset of sexual maturation. Guppies showed the ability to solve the cylinder task at all ages tested. However, their performance (i.e., number of correct trials) varied during development. The younger guppies (10 days old) were more likely to commit errors and touch the transparent glass compared to the two intermediate age groups (20 and 40 days). Unexpectedly, the older group of guppies showed a decline in performance. In a second study, guppies showed the capacity to solve the tube task even at the age of 5 days (Montalbano et al. 2023). In contrast with the study with the cylinder task, the performance of guppies in the tube task increased after reaching sexual maturation (e.g., age: 120 days). It is clear that in guppies’ IC is present at birth, although its developmental trajectory is not consistent across the two tasks. The contrasting results obtained in the older age groups of guppies might be related to sexual maturation and require investigations into the effect of sexual hormones on IC. Sexual hormones often determine adaptive changes in cognitive functioning (Ziomkiewicz et al. 2019). Alternatively, it is worth considering a potential role of changes in motivation. The older guppies of the study by Montalbano and colleagues (2023) were also significantly faster at solving the task, suggesting that they could have been more motivated to reach the food. This higher motivation may have reduced their inhibitory performance.

Montalbano and colleagues (2023) also investigated IC across development in another species, the zebrafish. Interestingly, they did not find the capacity to solve the tube task before an age of approximately 60 days, in which zebrafish are usually considered juveniles (Montalbano et al. 2023). There were no age differences in a measure of motivation (i.e., latency to approach the food) that could explain the result. In zebrafish, hatching occurs at a very early stage of embryonic maturation, approximately 40–72 h after the fertilisation. Conversely, the guppy is a live-bearing species and newborns are fully developed after a gestation of approximately 30 days. It was therefore suggested that the developmental mode of the species determines the developmental trajectory of IC. An alternative hypothesis worth investigating is related to eventual ecological shifts during the development (Takahashi et al. 2010). The variability in the ecology and the reproductive mode of teleost fish may be both important to determine evolutionary differences in trajectories of cognitive development, a topic that has received relatively little attention in the field of animal cognition.

One study has investigated the age-related variation in IC after sexual maturation in eastern mosquitofish (Vinogradov et al. 2022). The subjects showed an increase in IC performance measured with the detour task across three age groups at 7, 14, and 21 weeks after maturation. This result is intriguing considering that in humans, IC continues to improve for few years after puberty, but then declines during aging (e.g., Chao and Knight 1997). The average life span of eastern mosquitofish has been reported to be 12–15 months for females, and less for males (Aich et al. 2021), although older individuals have been commonly sampled in the field (reviewed in Cheng et al. 2018). It would be interesting to investigate IC in much older fish subjects to assess the presence of an aging-related decrease.

Recent studies have shed light on the ontogenetic influence of the environment on IC, indicating phenotypic plasticity in this cognitive function, particularly during early developmental stages. For instance, guppies raised in stable social groups developed enhanced IC compared to guppies raised in unstable social groups (Lucon-Xiccato et al. 2022a). This suggests that living in socially stable groups with clear hierarchies demands greater inhibition. The study also found that guppies raised as single individuals have greater inhibitory control compared to those raised in pairs or in groups, albeit this effect was less clear. Another study reported that guppies raised in an unpredictable environment, where the timing and location of food resources changed continuously, developed greater IC compared to guppies exposed to predictable food resources (Lucon-Xiccato et al. 2023). This plasticity might be adaptive, as inhibition is likely advantageous in dynamic and unpredictable situations. In line with this interpretation, the unpredictable treatment decreased learning performance. Another study on plasticity showed that exposing developing guppies to environments with variations in biotic and abiotic stimuli (e.g., environmental enrichment) altered learning but not IC (Montalbano et al. 2022). Together with the results from the previous study, the work by Montalbano and colleagues (2022) suggests that certain forms of cognitive plasticity are function-specific and directly influence IC, potentially offering selective advantages in specific environments. Overall, the developmental trajectory of guppies IC appears to be highly plastic, influenced by several factors (and their interaction) experienced by individuals. Further exploration of this plasticity in other fish species is warranted.

## Conclusions

**Fig. 4:**
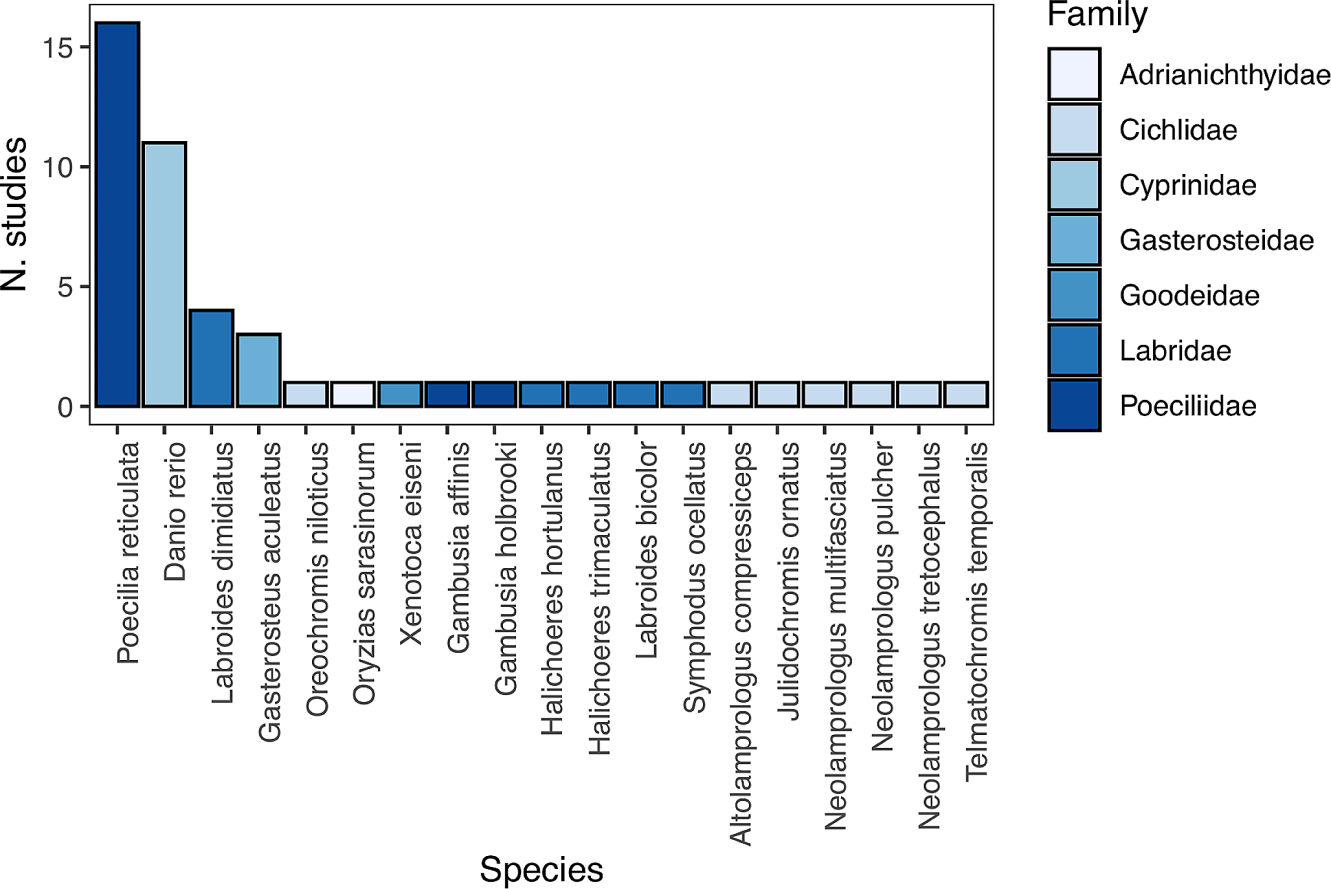
Number of studies on inhibitory control of fish in the literature divided by the species investigated. Colours indicate families

This review underscores the growing significance of research on IC in fish over recent years. Within this review, data were recovered from 20 teleost species belonging to eight families (Fig. 4), which is a relatively high taxonomic coverage for a field in animal cognition still in its early stages. However, it is essential to note that a predominant focus on few reference species exists within the literature. The guppy and the zebrafish alone contribute to over half of the experiments on IC in fish (Fig. 4). This means that we have significant data on IC only for these two species. Conversely, over half of the remaining species are represented by a single study, impeding the generalization of the findings. For instance, research on ecological specialisation has predominantly centred around the peculiar foraging behaviour of the cleaner wrasse (e.g., Aellen et al. 2021; Danisman et al. 2010), from which it is not possible to infer general patterns appliable to other species. It is critically important to broaden IC research in fish by incorporating a more extensive array of species and higher taxa. A positive example is the study of sex differences, involving at least six teleost species to date. It is noteworthy that, to the best of my knowledge, the literature lacks studies on cartilaginous fish, even if this group garners increasing attention for other cognitive abilities (reviewed in Brown and Schluessel 2023).

The availability of several IC paradigms provides a notable advantage in fish research. In contrast, for other executive functions, such as cognitive flexibility, only one established paradigm exists (e.g., the reversal learning task). The most widely used IC tests are based on fish spontaneous behaviour (Fig. 2b) and are therefore, well-suited for large sample sizes. Perhaps for this reason, a substantial portion of the literature in fish has focussed on intraspecific variability in IC, a field that is challenging to address with the typical small samples of training procedures. In studies requiring large phenotypic screenings, fish might have an advantage over other vertebrate species. However, at least two methodological limitations have emerged from this review. First, most of the studies have exploited paradigms based on transparent barriers (Fig. 2b). While this preference aids comparison with earlier data for other species, it certainly limits the information that we can obtain and it is susceptible to the issues reported for this type of methodology (e.g., Santacà et al. 2019b; van Horik et al. 2018). The second methodological limitation observed is that some simple paradigms adopted to study IC in other species have not yet been developed for fish. Among the others, the A-not-B task and the go-no-go task (e.g., Fagnani et al. 2016; Wessel 2018) appear the easiest to implement.

From a conceptual point of view, the literature reviewed has highlighted a relatively broad coverage of topics with important findings. Studies examining IC of fish species with a comparative perspective suggest similarities both at the performance and the mechanism levels, with the more studied vertebrate groups, namely birds and mammals. Research in fish has also identified all the main forms of intraspecific variation in IC known for warm-blooded vertebrates. This emphasises the potential of fish to provide valuable insights into questions on the evolution of IC. However, in many contexts, robust conclusions have not been reached so far, necessitating further research efforts. Additionally, there are entire aspects of IC that remain unexplored in fish. Most of the paradigms focus on inhibition of motor responses. Self-control, a form of IC that enables individuals to resist immediate small rewards to gain larger rewards in the future, has received surprisingly little attention in fish, despite being routinely investigated in other vertebrates (e.g., Miller et al. 2019). Due to possible publication biases, it is difficult to exclude that some research groups have actually attempted to measure self-control in fish without success. Similarly, there has been no direct testing of attentional inhibition.

Another literature gap that warrants attention is the limited understanding of molecular mechanisms underpinning IC in fish. Despite that fish are considered paramount models in applied research to investigate cognitive mechanisms, few steps have been taken towards this direction in IC research. Contemporary research technologies allow, for instance, relatively simple and cost-effective genetic manipulation in species such as the zebrafish and the medaka (e.g., Gibert et al. 2013; Norton and Bally-Cuif 2010; Stewart et al. 2014). However, such genetic manipulation was done only in one study for IC research. Moreover, in zebrafish, it is possible to investigate cognitive substrates using live imaging techniques with single-neuron resolution throughout the entire brain (Cong et al. 2017). Yet, only one study has investigated IC brain substrates and with a limited resolution. A crucial objective of future research in fish is to apply the available methodologies to study the molecular and neural mechanisms of IC.

Last, although beyond the scope of the present review, it is worth mentioning that knowledge on fish IC may, in the future, contribute to the development of models for health and social issues related to IC disorders in humans. For example, there is a high incidence of eating disorders and drug misuse in people with low IC (Colzato et al. 2007; Fogel et al. 2019; López-Caneda et al. 2013) and Alzheimer’s disease (Crawford et al. 2005) as well as attention deficit/hyperactivity disorders (ADHD) are characterised by a reduction of IC (Schachar et al. 2000). Fish species, such as the zebrafish, are increasingly used as models for behavioural and cognitive disorders (reviewed in Best and Alderton 2008; Kalueff et al. 2014), especially due to the aforementioned tools for studying brain mechanisms (e.g., Norton and Bally-Cuif 2010). These models are particularly useful for testing and developing new pharmacological therapies (reviewed in Goldsmith 2004; Langheinrich 2003). Some studies have begun to explore the use of fish models for research on IC disorders. For instance, the effects of a few pharmaceutical drugs have been characterised (Parker et al. 2014). However, more research efforts in this direction are necessary.

## Data Availability

Data used for plots are freely available following the reference provided in the text.
